# High Density Supercritical Carbon Dioxide for the Extraction of Pesticide Residues in Onion with Multivariate Response Surface Methodology

**DOI:** 10.3390/molecules25041012

**Published:** 2020-02-24

**Authors:** Teshome Tolcha, Tura Gemechu, Said Al-Hamimi, Negussie Megersa, Charlotta Turner

**Affiliations:** 1 Department of Chemistry, Addis Ababa University, P. O. Box 1176 Addis Ababa, Ethiopia; bonisalale@gmail.com (T.T.); turagemechu2006@gmail.com (T.G.); negussie.megersa@gmail.com (N.M.); 2 Department of Chemistry, Lund University, Centre for Analysis and Synthesis, P. O. Box 124, SE-22100 Lund, Sweden; said.alhamimi@chem.lu.se

**Keywords:** density, onion, organochlorine, pesticide residues, *s*-triazine, supercritical fluid extraction, ultrahigh pressure

## Abstract

The excessive use of pesticides is a serious health problem due to their toxicity and bioaccumulation through the food chain. Due to the complexity of foods, the analysis of pesticides is challenging often giving large matrix effects and co-extracted compounds. To overcome this problem, a selective and “green” supercritical fluid extraction method was developed, using neat carbon dioxide as a solvent at pressures of up to 800 bars. A Box–Behnken response surface experimental design was used, with the independent variables of density (0.70−1.0 g mL^−1^), temperature (40−70 °C), and volume (10−40 mL) of solvent, and the dependent variable of extracted amount of pesticides. The optimum extraction condition was found at the use of 29 mL of supercritical CO_2_ at 0.90 g mL^−1^ and 53 °C (corresponding to 372 bars of pressure). It was observed that increasing the density of CO_2_ significantly increased the extraction recovery of endrin and 2,4*′*-dichlorodiphenyldichloroethane. Matrix-matched calibration curves showed satisfactory linearity (*R*^2^ ≥ 0.994), and LODs ranged from 0.2 to 2.0 ng g^−1^. Precision was lower than 11% and recoveries between 80%–103%. Thus, the developed method could efficiently be used for trace analysis of pesticides in complex food matrices without the use of organic solvents.

## 1. Introduction

The use of chemical pesticides in modern agriculture to ensure food security has increased over the past decades [1,2]. Organochlorine insecticides and *s*-triazine herbicides for effective control of a variety of insects and weeds are commonly used in developing countries [3,4]. The extensive application of these compounds and their degradation products contaminate the environment and agricultural products [5,6]. Moreover, water-soluble compounds are transported to surrounding water and eventually enter the food chain [7,8]. Lipophilic compounds like organochlorine can also concentrate in fatty tissues and bioaccumulate in the food chain [2]. 

Although applications of organochlorine and *s*-triazine pesticides have been banned in developed countries, developing countries like Ethiopia are still using them for agricultural and medicinal purposes. Studies carried out in various agro-industries in Ethiopia showed the presence of contaminant pesticide residues in different samples collected from various localities of Ethiopia [6]. The obtained results revealed the presence of 4,4’-dichlorodiphenyltrichloroethane (4,4´-DDT) in *Khat*, diazinon in *Khat* and wheat [9], 4,4´-DDT, 4,4-dichlorodiphenyldichloroethylene (4,4-DDE) and 4,4*′*-dichlorodiphenyldichloroethane (4,4´-DDD) in human and cow milk [10], and 4,4-DDTs in fish [6].

Pesticide pollutants are found at trace levels, and samples from agricultural products are complex in nature. Thus, selective and efficient sample preparation methods that can extract and preconcentrate prior to their instrumental determination are needed [11,12,13]. Traditionally, the determination of pesticide residues has relied on the use of liquid–liquid extraction (LLE) and solid-phase extraction (SPE) [14]. However, LLE suffers from certain drawbacks including difficulty of automation, high consumption of toxic organic solvents and an often tiresome formation of emulsions [15,16,17]. On the other hand, SPE requires column conditioning, elution with organic solvents, and the technique is not appropriate for multi pesticides analysis [18]. Microwave-assisted extraction (MAE) and pressurized liquid extraction (PLE) have also been utilized for the extraction of pesticide residues from semi-solid and solid complex food matrices. The use of organic solvents and low selectivity are the major disadvantages of these extraction methods [19,20]. 

Recently, more attention has been given to the development of green extraction methods based on pressurized hot water extraction (PHWE) [21] and supercritical fluid extraction (SFE) [22]. In this regard, supercritical carbon dioxide (sc-CO_2_) is an interesting solvent due to its properties such as low viscosity and high diffusivity, which allows for faster extractions of mainly non-polar compounds [23]. Its high diffusivity and low viscosity allow efficient wetting and penetration into the solid matrix. This characteristic promotes more efficient extraction of the compounds as compared to conventional liquid organic solvents. The high achievable density of sc-CO_2_ gives the fluid a high solvation power of non-polar and medium-polar compounds, with high selectivity towards the unwanted more polar compounds [24]. SFE based on neat sc-CO_2_ is a green method, resulting in clean extracts and providing an inert environment for the analytes on the contrary to conventional solvent extraction [25]. However, SFE of pesticides from food samples generally requires the addition of co-solvents like methanol [22,26] or acetone [27] to overcome strong solute-matrix interactions. SFE with methanol as a co-solvent has been used for the extraction of dieldrin from radishes, chlorpyrifos from grass field samples, methamidophos from spiked vegetables, organophosphorus compounds from rice, carbendazime from lettuce leaves, fonofos from onions, pirimiphos methyl from wheat and beans, and atrazine from canola [22]. SFE with acetone as a co-solvent was also used for extraction of vinclozolin, tolclofos-methyl, malathion, chlorpyrifos, 4,4’-dichlorobenzophenone, procymidone, and 4,4’-DDE from strawberries. Furthermore, SFE at pressures of 120–350 bar has been applied for the extraction of different pesticides from fruits and vegetables [27].

**Figure 1:**
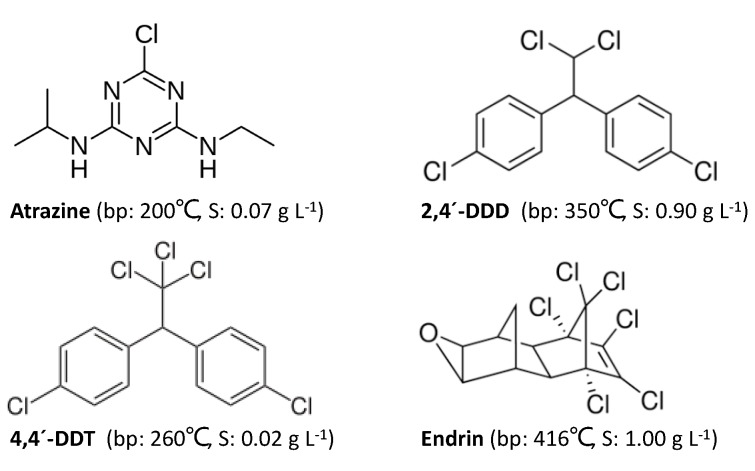
Chemical structures and physical constants (bp: boiling point; S: solubility in water) of the pesticides investigated in this study.

The aim of this study is to explore the effect of density and temperature of sc-CO_2_ on the extractability of pesticides at pressures of up to over 800 bars. It is the first time that an ultrahigh pressure (UHPSFE) method is applied for pesticide extraction from foods. The effect of density and temperature on solvation power of sc-CO_2_ for extractability of the selected pesticides atrazine, 2,4´-DDD, 4,4´-DDT and endrin (Figure 1) from onion samples were investigated. In order to maximize the pesticides extraction efficiency, a Box–Behnken response surface design (BBD) using a MODDE 10.1 software was used. BBD is useful to avoid experiments that are in extreme conditions because the highest level and lowest level combinations for every factor are not included [28].

## 2. Results and Discussion

In this study, what is unique is that ultrahigh pressure SFE (UHPSFE) is used for the extraction of pesticides, atrazine, 2,4´-DDD, 4,4´-DDT and endrin, from a food sample, in this case onion. It is well-known that sc-CO_2_ of very high pressure and density enable extraordinary solubility or even complete miscibility of some lipids [29]. In our previous research, we have demonstrated that Moringa oleifera and Moringa peregrina seed lipids could be efficiently extracted using UHPSFE, giving a clean oil extract without any solvent residues [30]. Results in the current study show that UHPSFE followed by analysis with GC/MS is a green method using minimal amounts of organic solvents.

### 2.1. Multivariate Parameter Optimization

A Box–Behnken experimental design was used to study three main independent variables: density, temperature, and total volume of supercritical carbon dioxide used during the extraction, which all could potentially affect the extractability of pesticides from onion. In the design of experiment (DoE), density was used rather than pressure, since the latter does not have an impact on the extraction efficiency, while density does. If pressure and temperature are used in a DoE, the density values will be non-linearly distributed between the experimental points, which is problematic. The total volume of extraction solvent was used in the DoE instead of using extraction time at a constant flow rate, which is due to the fact that the flow rate was difficult to control at the high pressures used in this study. The experimental design chosen for the optimization provides a reduced number of experiments (15 as compared to 27 (3^3^) experiments using a full factorial design) without loss of significant information [28]. The investigated range for each variable and the obtained responses in each experiment are given in Table 1 and Table 2, respectively. The investigated ranges were selected based on the limitation of the extraction instrument. Other parameters implicated in the extraction were kept constant, i.e., the amount of dried sample (1.0 g) and flow rate (3.0 mL min^−1^). Each experiment was done by spiking 1.0 g of onion sample at 500 µg kg^−1^ concentration level.

The detected peak area for each compound in the experiments (based on GC/MS analysis) was converted to recovery and treated as a response in the design. Figure S1 indicates the summary of model fit using Multinomial Logistic Regression (MLR). The high values for *R*^2^ and *Q*^2^ indicate that the quadratic equation well represents the system under the given experimental domain. All compounds gave valid models since all *R*^2^ are greater than 0.9 and *Q*^2^ are greater than 0.65. It is also evident from the fact that the linearity plot depicted in Figure S2 indicates a satisfactory correlation between the observed and predicted recovery of all target pesticides. The data points clustering around the diagonal line show a good fit of the model.

**Figure 2:**
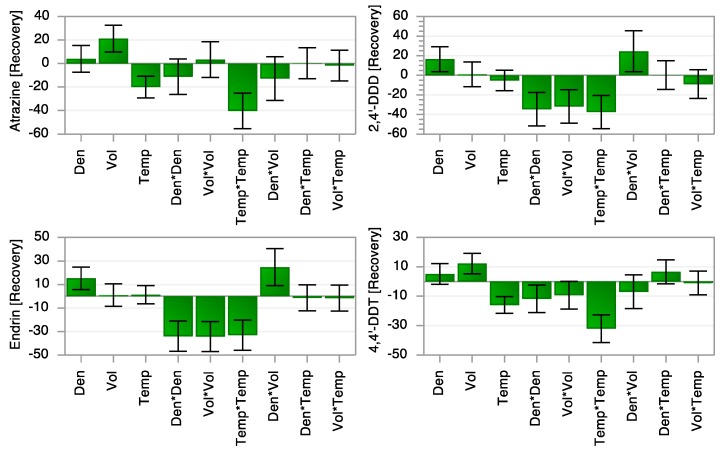
Effect of extraction variables and their interactions on the recovery (%) of analytes. Den, Vol and Temp represent density, volume and temperature, respectively.

The coefficient plot in Figure 2 shows the effect of individual variables and their interactions on the extraction efficiency of pesticides from an onion sample. The figure clearly indicates that the density of sc-CO_2_ has a positive and significant effect on the extraction recovery of 2,4´-DDD and endrin. For both compounds, this could be explained by the fact that these two compounds are more polar than the others (see Figure 1) and that the polarity of sc-CO_2_ increases slightly with density. Furthermore, the extraction temperature has a negative and significant effect upon the extraction efficiency of atrazine and 4,4´-DDT. A likely explanation is that these two compounds have the lowest boiling point of the four compounds investigated here, which means that they are more prone to losses during the collection step of UHPSFE. It has been previously shown that collection in SFE may introduce negative bias [31].

**Figure 3:**
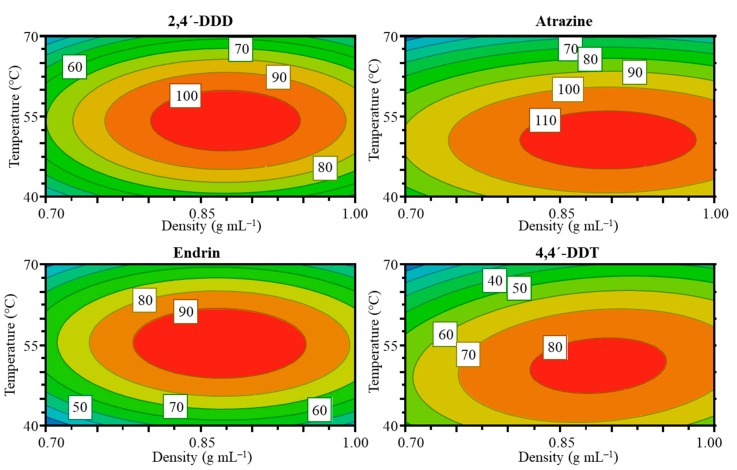
Response surface contour plots for analyte recoveries (%) vs. the extraction variables temperature (°C) and density (g mL^−1^). Contour plots for volume/density and temperature/volume are found in the Supporting Information.

The regression models obtained were used to calculate the response surfaces as contour plots for each variable separately (Figure 3 and Figure S3). Figure 3 shows response contour plots for the analyte recoveries, and the plots given were used for interpreting the variations of recovery as a function of each pair of the independent variables. 

From the graph in Figure 3, the interaction of temperature and density can be seen; the extractability of endrin and to some extent also 2,4’-DDD is assisted by an increasing temperature, while an increasing density has a positive effect for the other analytes. This is logic, considering the higher boiling point of endrin, followed by 2,4’-DDD, and then 4,4´-DDT and atrazine. An optimizer function based on a simplex algorithm with a non-linear desirability function was applied to find the conditions where the highest responses are existing, giving equal weigh for all analytes. It turned out that sc-CO_2_ density of 0.90 g mL^−1^ at 53 °C (372 bars of pressure) and 29 mL of sc-CO_2_ was the optimum extraction condition for maximum recoveries. 

### 2.2. Analytical Performance of the New Extraction Method

The proposed method was evaluated in terms of linear range, LODs, precision, and accuracy using onion samples spiked with the analytes under study. The obtained results are summarized in Table 3. Matrix-matched calibration curves were established using onion samples spiked at five different concentration levels (7.8, 125, 250, 500, and 2000 μg kg^−1^) for all analytes except endrin (31.2, 125, 250, 500, and 2000 μg kg^−1^) and treated following the developed sc-CO_2_ extraction procedure.

The results show that the proposed method is linear with *R^2^* ranging between 0.994–0.999. LODs ranging between 0.2 and 2.0 ng g^−1^ were achieved. Precision studies were carried out in order to evaluate the repeatability (intra-day precision) and reproducibility (inter-day precision) of the proposed sc-CO_2_ extraction with the GC-MS detection method. Repeatability and reproducibility were assayed by analyzing spiked samples at 500 μg kg^−1^ concentration levels. Results expressed as relative standard deviation of response (%RSDs) are shown in Table 3, demonstrating values lower than 11%.

**Figure 4:**
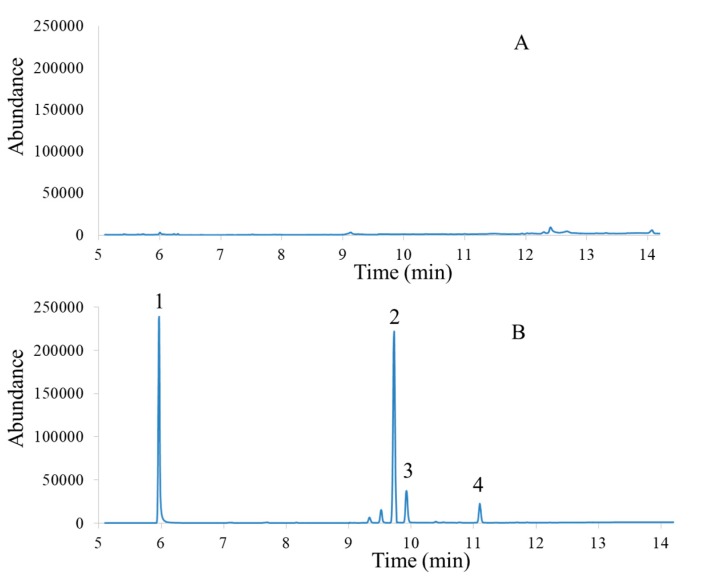
Typical chromatograms of unspiked (**A**) and spiked (**B**) onion extract. Experimental condition: Sample amount, 1.0 g; spiking level, 500 μg kg^−1^; flow rate, 3.0 mL min^−1^ density of sc-CO_2_, 0.90 g mL^−1^; volume of sc-CO_2_, 29 mL and extraction temperature, 53 °C. Peaks: 1, atrazine; 2, 4,4’-DDE; 3, endrin; 4, 2,4’-DDT.

In order to check the trueness of the proposed method, recovery experiments were performed using spiked onion sample at 500 μg kg^−1^ concentration level. Recoveries were estimated by the comparison of the obtained signal for each analyte with the signal obtained for a blank sample spiked after the sample treatment and prior to its analysis. As can be seen, recoveries range from 80 to 103%, demonstrating the convenience of the proposed sample pre-treatment for quantitative and qualitative analysis of pesticide residues studied in onion samples [32]. Moreover, a blank onion sample was analyzed and there was no co-elution observed for the pesticides under investigation (Figure 4). These results could further be used as a basis to draw the conclusion that the matrices of the onion samples do not have significant effects on the proposed method for extraction of the analytes. The experimental findings reveal that the onion sample tested is either free of the pesticides investigated, or the levels are below the detection limit of the proposed analytical method.

### 2.3. Comparison of the New SFE Method with Other Extraction Methods in the Literature

The proposed extraction method, SFE using neat ultrahigh pressure sc-CO_2_ as a solvent followed by GC-MS analysis, was compared with solid phase extraction followed by gas chromatography with electron capture detector (SPE with GC-ECD) [33], a quick, easy, cheap, effective, rugged, and safe extraction method followed by GC with pulsed flame photometric detector (QuEChERS with GC-FPD) [34], QuEChERS with multi-walled carbon nanotubes (MWCNTs) with GC-MS analysis [35], and QuEChERS with GC-MS analysis [36]. The summary of the results obtained is given in Table 4. Compared to these methods, the proposed method provided among the highest recoveries and lowest LOD, as well as a better or comparable coefficient of determination. In addition, the new method is likely to be greener compared to the other analytical methods because it uses only sc-CO_2_ as an extraction solvent. Hence, the proposed method is a promising extraction method for pesticides having relatively similar or slightly different physical and chemical properties.

## 3. Materials and Methods

### 3.1. Chemicals and Reagents

Ultrapure CO_2_ in cylinders with a dip tube was provided by AGA industrial gases (Lidingö, Sweden). Methanol and *n*-heptane of chromatographic grade were purchased from VWR BDH Chemicals (Gdańsk, Poland) and Fisher Chemicals (Loughborough, UK), respectively. Analytical grade standards including atrazine, 2,4´-DDD, 4,4´-DDT and endrin were purchased from Sigma-Aldrich (Buchis, Switzerland). The stock solution was prepared by weighing 2.5 mg of each standard and dissolving in a small amount of ethyl acetate, and the resulting solution was diluted with methanol in a 25 mL volumetric flask and stored at −20 °C. Mixed working standard solutions, 10 mg L^−1^, were prepared by proper dilution of the stock solution with methanol once per week. This working solution was used during method development and validation. A 1.0 g onion sample was spiked at 500 µg kg^−1^ for each pesticide and air dried at room temperature for 24 h before extraction in order to achieve as much adsorption of the pesticides to the sample matrix as possible. 

### 3.2. Sample Site and Sampling 

The onion sample was collected randomly from a local market in Addis Ababa, Ethiopia. The geographical location of the sampling site is 8°58’50.17’’ N and longitude 38°48’27.94’’ E with elevation of 2296.1 m above sea level. The onion was cut into small pieces using an iron knife and was thereafter air dried. It was further dried in oven at 92 °C (boiling point of water in Addis Ababa) until constant mass was obtained. Then, the sample was ground with an electric mill, sieved through a 0.25 mm pore size, wrapped in a methanol-rinsed aluminum foil, and kept in a polyethylene plastic bag.

### 3.3. Multivariate Experimental Design

A Box–Behnken response surface experimental design was used to optimize independent quantitative variables including density (0.70–1.0 g mL^−1^), volume (10–40 mL) and temperature (40–70 °C). The design was created using MODDE 10.1 software package (Sartorius Stedim Biotech, Malmö, Sweden) and the responses for each experiment were calculated based on the recovery. The density of sc-CO_2_ was calculated using NIST Chemistry WebBook, SRD 69 (Gaithersburg, MD, USA) and given in Table S1. The adequacy of the models was evaluated by the *R*^2^ and *Q*^2^ values (where *R*^2^ shows the model fit and *Q*^2^ shows an estimate of the future prediction precision), predicted vs. observed plot and coefficient plots. *Q*^2^ should be greater than 0.1 for a significant model and greater than 0.5 for a good model. The optimum processing conditions for the maximum peak area for each target compound were obtained by using graphical and numerical analysis based on the criteria of the desirability function and the response surface plots.

### 3.4. Supercritical Fluid Extraction Procedure

Supercritical fluid extraction (SFE) of pesticides was carried out by using a home-built extraction system consisting of an ISCO 65D syringe-pump (Teledyne Isco, Thousand Oaks, CA, USA), a GC oven in which the extraction vessel was placed, and a needle valve connected to a restrictor (a long stainless-steel tube with internal diameter 0.0036 inch) to control the flow rate. A 1.0 g onion sample was spiked by adding an appropriate volume of a standard working solution and mixed with 1.0 g of glass beads to reduce the volume into a 5 mL stainless-steel extraction vessel in sandwich mode, using 3 mm glass beads at both the bottom and the top of the cell to protect the cell sealing. Then, it was heated in the GC oven for 5 min at 53 °C and extraction was continued in a dynamic mode using 29 mL of CO_2_ at 3 mL min^−1^. The density of sc-CO_2_ was kept at 0.90 g mL^−1^. Then the extraction was flushed with 2 mL of *n*-heptane and dried under a gentle N_2_ stream. Finally, the extract was reconstituted with 100 µL of *n*-heptane, and 2 µL of this was injected to GC-MS for analysis. A calibration curve was achieved by extracting onion samples spiked at five different concentration levels (7.8, 125, 250, 500, and 2000 μg kg^−1^) for all analytes except endrin (31.2, 125, 250, 500, and 2000 μg kg^−1^). Repeatability and reproducibility were studied by extracting onion spiked with a mixture of all analytes at 500 μg kg^−1^ following the optimized extraction parameters.

### 3.5. GC-MS Analysis

A HEWLETT G2614A gas chromatography (GC) equipped with G1099A mass spectrometry (MS) (Hewlett Packard, Palo Alto, CA, USA) was used for analysis and quantification. A HP-5 ms (USA) ultra-inert capillary column (30 m × 250 µm and 0.25 µm i.d.) was used for separations. Helium gas of purity 99.999% was used as carrier gas at a flow of 1.0 mL min^−1^. The oven temperature program used during analysis was set as follows: 82 °C for 1 min; increased to 185 °C at 25 °C min^−1^ ramp rate and held at this temperature for 1 min; then increased to 250 °C at 9 °C min^−1^ ramp rate and maintained at this temperature for 1 min. The GC injection port temperature was kept at 250 °C. Splitless injection mode was used during the whole analysis. Peaks were identified by their retention time and mass spectra after acquisition of the total ion chromatogram. In order to confirm the retention times of all analytes, scan mode was carried over the range 50–550 *m/z*. Selective ion monitoring (SIM) of each pesticide and two characteristic fragment qualifier ions were selected to identity of pesticides. The *m/z* selected for SIM mode detection was: atrazine (173, 200, 215), 2,4´-DDD (199, 235, 237), 4,4´-DDT (212, 235, 239), and endrin (245, 263, 281). Peak area was utilized as an instrumental response, which was converted later to recovery. Recovery for each pesticide was calculated using the concentration obtained after extracting spiked onion following the optimum extraction concentration.

### 3.6. Method Validation

In order to validate the proposed analytical method, linearity, precisions, and limits of detection (LOD) were evaluated. The linearity of the method was done at five different concentration levels, 7.8, 125, 250, 500, and 2000 μg kg^−1^ for all analytes except endrin, which was calibrated at 31.2, 125, 250, 500, and 2000 μg kg^−1^. Each SFE experiment was repeated in triplicate following the optimized procedure and injected in into GC-MS in triplicate. Repeatability (intra-day precision) and reproducibility (inter-day precision) of the method was evaluated using onion samples spiked with each pesticide at 500 μg kg^−1^. Recovery analysis was done for each analyte following the same procedure and the same concentration. The limit of detection (LODs) was determined as the lowest concentration yielding a signal to noise (S/N) ratio of 3 [37].

## 4. Conclusions

An UHPSFE method with GC-MS analysis was developed for the determination of four pesticide residues in onion samples. The extraction variables density, temperature and volume of sc-CO_2_ were optimized by a Box–Behnken design, achieving high analyte recoveries, low LODs and good precision. The results also indicate that the variables have a marked influence on the extraction recoveries, but are different for the different analytes. For instance, a higher density of the extraction solvent (sc-CO_2_) was beneficial for the relatively polar pesticides. The optimized method was successfully applied to the analysis of atrazine, 4,4’-DDE, endrin and 2,4’-DDT residues in spiked onion samples. In comparison to extraction methods in the literature, the new proposed method provides a solvent-free green alternative.

## Figures and Tables

**Table 1:** Surface response Box–Behnken experimental design of variables for optimization of SFE method.

Parameters	N1	N2	N3	N4	N5	N6	N7	N8	N9	N10	N11	N12	N13	N14	N15
Density (g mL^−1^) (Pressure (bar))	0.7 (168)	1.0 (668)	0.7 (168)	1.0 (668)	0.7 (114)	1.0 (528)	0.7 (223)	1.0 (807)	0.85 (211)	0.85 (211)	0.85 (388)	0.85 (388)	0.85 (300)	0.85 (300)	0.85 (300)
Volume (mL)	10	10	40	40	25	25	25	25	10	40	10	40	25	25	25
Temperature (°C)	55	55	55	55	40	40	70	70	40	40	70	70	55	55	55

N: Experiment number.

**Table 2:** Recovery (%) of the analytes obtained using a Box–Behnken DoE.

Pesticides	N1	N2	N3	N4	N5	N6	N7	N8	N9	N10	N11	N12	N13	N14	N15
Atrazine	71	102	100	117	78	88	38	49	74	122	36	78	102	113	128
2,4´-DDD	41	30	59	87	23	49	17	45	37	52	40	17	93	114	110
Endrin	43	19	61	65	11	49	14	47	23	33	31	34	89	107	97
4,4´-DDT	32	59	34	72	52	46	12	33	45	69	10	29	74	82	83

**Table 3:** Analytical performances of the proposed SFE method with GC-MS analysis for onion samples spiked with atrazine, 2,4’-DDD, endrin, and 4,4’-DDT.

Pesticide	Linearity (µg kg^−1^)	(R^2^)	Predicted Recovery	Experimental Recovery (%) (%RSD)	Repeatability (%RSD)	Reproducibility (%RSD)	LOD (µg kg^−1^)
Atrazine	7.8–2000	0.999	121	93 (4.8)	3.8	1.8	0.2
2,4’-DDD	7.8–2000	0.998	108	93 (3.3)	0.9	3.6	0.4
Endrin	31.2–1000	0.998	98	103 (3.7)	10.2	8.3	2.0
4,4’-DDT	7.8–2000	0.994	82	80 (3.2)	4.4	2.9	0.6

**Table 4:** Comparison of the new method with published methods found in the literature.

Method	Pesticides	Matrix	Recovery (%)	LODs (µg kg^−^^1^)	*R* ^2^	Ref.
SFE, GC-MS	Organochlorines and s-triazines	Onion	80–103	0.2–2	0.997–0.999	This study
SPE, GC-ECD	Organochlorines and pyrethroid	Fruit, vegetables	54–104	0.3–15	0.998–0.999	[33]
QuECheRS, GC-FPD	Organophosphorus	Onion	61–105	2–10	-	[34]
QuEChERS, MWCNTs, GC-MS	Multiclass pesticides	Leek, onion, ginger, garlic	78–110	2–20	>0.99	[35]
QuEChERS, GC-MS	Organochlorine, organophosphate and pyrethroid	Wheat grains, flour, bran	70–120	>2.5	0.99–1.0	[36]

## Supplementary Materials

The following are available online at https://www.mdpi.com/1420-3049/25/4/1012/s1, Figure S1: Summary of model fit; Figure S2: The linearity plot of predicted versus observed recovery (%); Figure S3: Response contour plot obtained from BBD for extraction variables and recoveries (%).
