# Supplementary material for: High Density Supercritical Carbon Dioxide for the Extraction of Pesticide Residues in Onion with Multivariate Response Surface Methodology
Source: Molecules. 2020 Feb 24;25(4):1012. doi: 10.3390/molecules25041012 (PMC7070919; doi:10.3390/molecules25041012)
Supplement: Supplementary file 1 [file molecules-25-01012-s001.pdf]

Supplementary Information

High Density Supercritical Carbon Dioxide for the Extraction of Pesticide Residues in Onion with Multivariate Response Surface Methodology

Teshome Tolcha [1], Tura Gemechu [1], Said Al-Hamimi [2], Negussie Megersa [1] and Charlotta Turner [2,*]

[1] Addis Ababa University, Department of Chemistry, P. O. Box 1176, Addis Ababa, Ethiopia; bonisalale@gmail.com (T.T.), turagemechu2006@gmail.com (T.G.), negussie.megersa@gmail.com (N.M.)
[2] Lund University, Department of Chemistry, Centre for Analysis and Synthesis, P. O. Box 124, SE-22100 Lund, Sweden; said.alhamimi@chem.lu.se
* Correspondence: Charlotta.Turner@chem.lu.se; Tel.: +46-46-222-8125

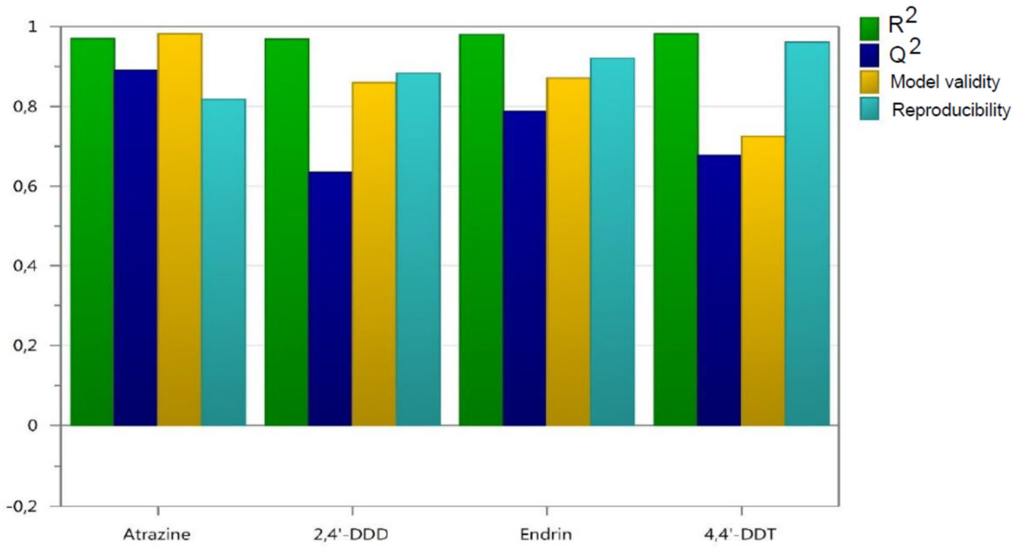

Figure 1. Summary of model fit.

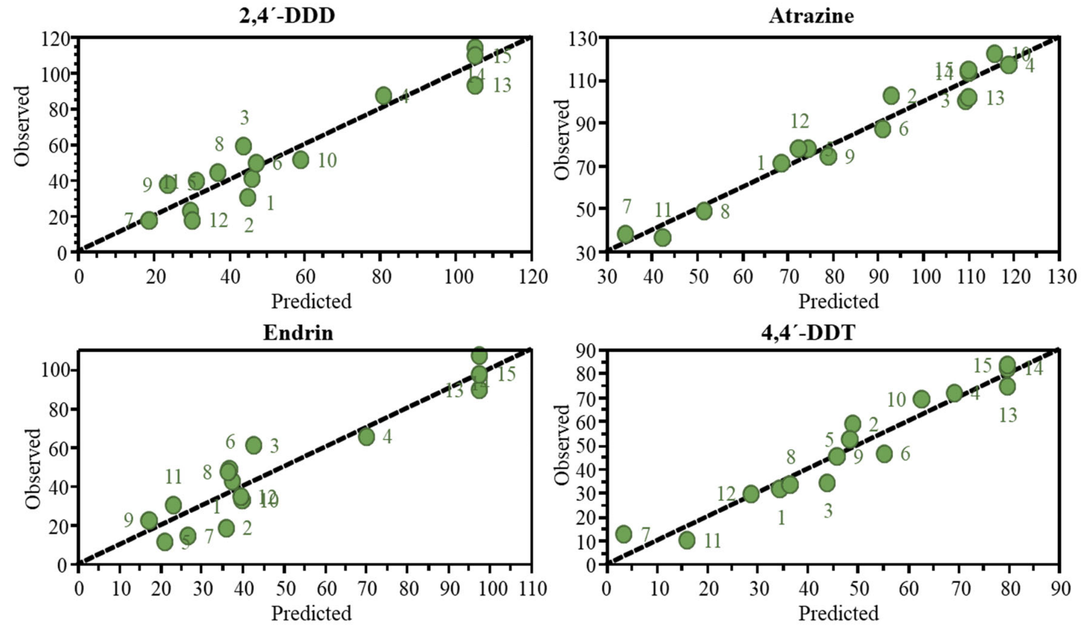

Figure 2. The linearity plot of predicted versus observed recovery (%).

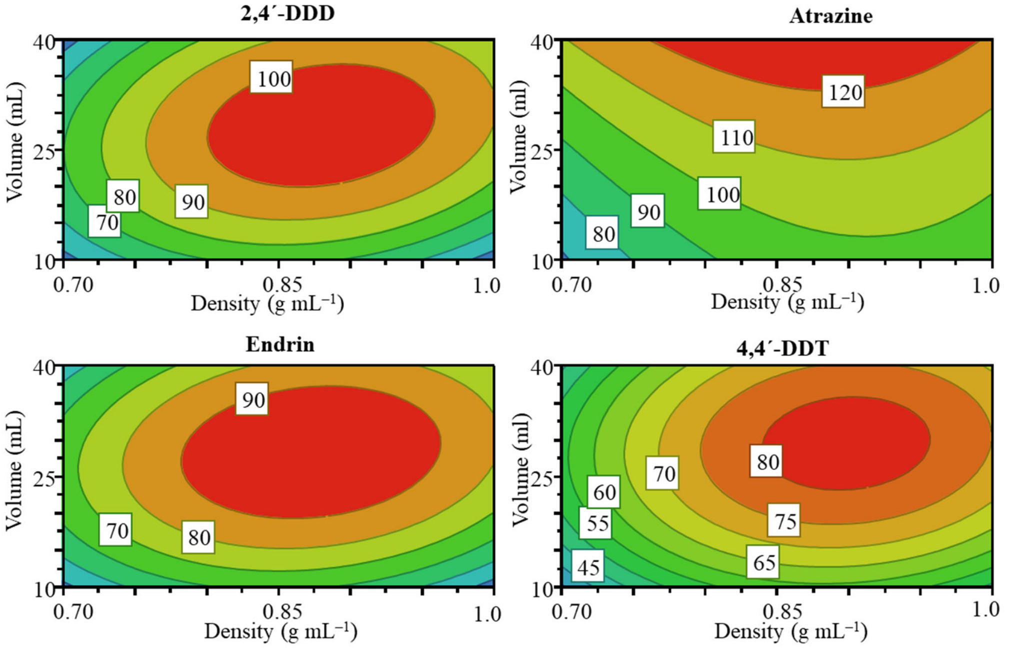

(**A**)

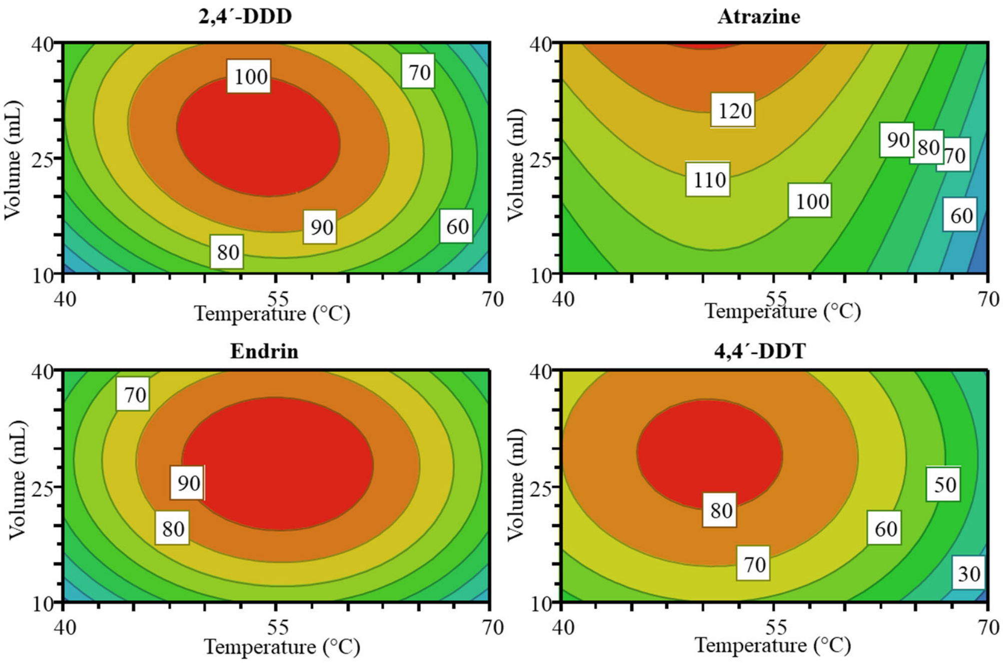

(**B**)

Figure 3. Response contour plot of volume versus density (A) and volume versus temperature (B) obtained from BBD for extraction variables and recoveries (%).
